# The Gut Microbiome and Female Health

**DOI:** 10.3390/biology11111683

**Published:** 2022-11-21

**Authors:** Ruqaiyyah Siddiqui, Zinb Makhlouf, Ahmad M. Alharbi, Hasan Alfahemi, Naveed Ahmed Khan

**Affiliations:** 1 College of Arts and Sciences, American University of Sharjah, University City, Sharjah 26666, United Arab Emirates; 2 Department of Medical Biology, Faculty of Medicine, Istinye University, Istanbul 34010, Turkey; 3 Department of Clinical Laboratory Sciences, College of Applied Medical Sciences, Taif University, Taif 21944, Saudi Arabia; 4 Department of Medical Microbiology, Faculty of Medicine, Al-Baha University, Al-Baha 65799, Saudi Arabia; 5 Department of Clinical Sciences, College of Medicine, University of Sharjah, University City, Sharjah 27272, United Arab Emirates

**Keywords:** gut microbiota, estrobolome, estrogen, probiotic, gut dysbiosis

## Abstract

**Simple Summary:**

A plethora of studies have highlighted the profound role of the gut microbiome in human health. However, there is a lack of studies on female health. Given that females may be more likely to be affected by some ailments such as osteoarthritis, heart disease, cancer, and anxiety, it is imperative to study the effect of the gut microbiome and its role in female health. It is evident that the presence/ratio of microbial species is altered in polycystic ovarian syndrome, cancer, pregnancy, and menopause. Thus, potential probiotics should be developed and the administration of certain bacterial species should be considered, as novel independent or adjunct therapies for various female-related pathologies. Strategies such as the modulation of the gut microbiome via diet and through supplementation with pre/pro/postbiotics in various female health-related issues should be undertaken.

**Abstract:**

The possession of two X chromosomes may come with the risk of various illnesses, females are more likely to be affected by osteoarthritis, heart disease, and anxiety. Given the reported correlations between gut microbiome dysbiosis and various illnesses, the female gut microbiome is worthy of exploration. Herein, we discuss the composition of the female gut microbiota and its dysbiosis in pathologies affecting the female population. Using PubMed, we performed a literature search, using key terms, namely: “gut microbiome”, “estrogen”, “menopause”, “polycystic ovarian syndrome”, “pregnancy”, and “menstruation”. In polycystic ovarian syndrome (PCOS), the abundance of *Bacteroides vulgatus, Firmicutes, Streptococcus,* and the ratio of *Escherichia/Shigella* was found to be increased while that of *Tenericutes* ML615J-28*, Tenericutes* 124-7*, Akkermansia, Ruminococcaceae,* and *Bacteroidetes* S24-7 was reduced. In breast cancer, the abundance of *Clostridiales* was enhanced, while in cervical cancer, *Prevotella, Porphyromonas,* and *Dialister* were enhanced but *Bacteroides, Alistipes,* and members of *Lachnospiracea,* were decreased. In ovarian cancer, *Prevotella* abundance was increased. Interestingly, the administration of *Lactobacillus acidophilus*, *Bifidobacterium bifidum, Lactobacillus reuteri,* and *Lactobacillus fermentum* ameliorated PCOS symptoms while that of a mix of *Bifidobacterium lactis* W51, *Bifidobacterium bifidum* W23, *Lactobacillus brevis* W63, *Bifidobacterium lactis* W52, *Lactobacillus salivarius* W24, *Lactobacillus acidophilus* W37, *Lactococcus lactis* W19, *Lactobacillus casei* W56, and *Lactococcus lactis* W58 alleviated vascular malfunction and arterial stiffness in obese postmenopausal women, and finally, while further research is needed, *Prevotella* maybe protective against postmenopausal bone mass loss. As several studies report the therapeutic potential of probiotics and since the gut microbiota of certain female pathological states has been relatively characterized, we speculate that the administration of certain bacterial species as probiotics is warranted, as novel independent or adjunct therapies for various female pathologies.

## 1. Introduction

An increasing body of research highlights the importance of gender differences in the epidemiology, pathophysiology, and treatment of various diseases, especially non-communicable diseases [1]. Furthermore, females are at a greater risk of suffering from osteoarthritis, heart disease, urinary tract problems, stroke, depression, and anxiety [2]. However, although females comprise almost half of the human population, there is a reported discrepancy in the presentation of the genders in health studies [3]. Moreover, women are underrepresented, and their physical complaints are trivialized [4,5]. Hence, many organizations call upon the inclusion of gender as a dimension in clinical trials [1]. Aside from obvious physical differences, the two genders are reported to have compositional differences in their gut microbiome [6]. Namely, a study employing 348 male and 341 female mice from 89 matched strains reported marked abundance differences among several taxa between the sexes, and a larger number of differences were observed at the single strain level [7]. A bidirectional relationship is established between host hormones and the host’s gut microbiome. Moreover, sex hormones can affect bacterial growth and virulence. Namely, the sex hormones estriol and estradiol inhibit bacterial virulence through hindering quorum sensing while progesterone was shown to enhance the growth of oral *Bacteroides* species and *Prevotella intermedius* [8]. Interestingly, some studies also imply that commensal microbiota can influence and modulate sex hormone levels [7]. While we still cannot define what a healthy gut microbiome is, gut microbial dysbiosis has been correlated with various diseases ranging from irritable bowel syndrome to cancer [9,10,11,12]. In light of the reported associations between the gut microbiome and overall human health, coupled with the disproportionately higher risk females are under for various diseases, the female gut microbiome is a topic worthy of investigation. Using PubMed as a database, we searched the literature using key terms, namely “gut microbiome”, “estrogen”, “menopause”, “polycystic ovarian syndrome”, “pregnancy”, and “menstruation” and a time interval ranging from 2012 to 2022 with a focus on recent studies. Herein, we review and describe the composition of the healthy female gut microbiome, the potential role of the gut microbiota and its dysbiosis in female diseases such as polycystic ovaries syndrome (PCOS) and female cancers, and otherwise, normal female physiology, namely, pregnancy, menstruation, and menopause, and finally the importance of the gut microbiota in postmenopausal health and postmenopausal illnesses.

## 2. Gut Microbiome in Healthy Females

**Figure 1:**
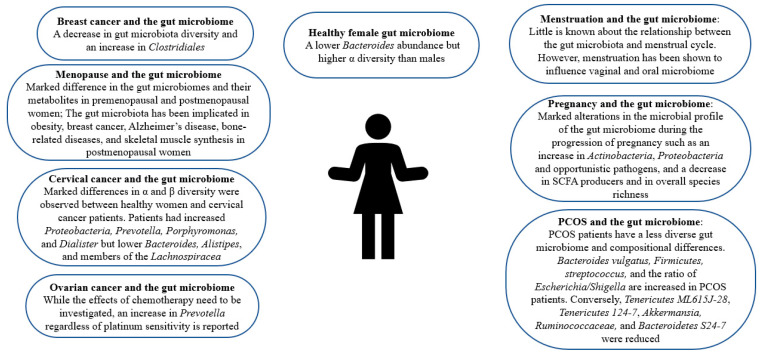
The role of gut microbiome in female health.

Gender-dependent differences in the gut microbiome have been reported [9]. Women’s gut microbiome composition markedly differs from men’s [13,14]. As depicted in Figure 1, women are reported to have a lower *Bacteroides* abundance but higher α diversity, a measure of diversity within an individual sample [13,14,15]. Female sex hormone levels affect the microbiota composition [16]. However, this influence is bidirectional. This is because the microbiome regulates steroid hormone levels including estrogens [17]. In a clinical trial employing 25 men, 7 postmenopausal women, and 19 premenopausal women, results imply that the intestinal microbiome indeed affects systemic estrogen levels [18]. Another study reports that gut microbial diversity is positively correlated with estrogen metabolites to parent estrogen ratio in a group of postmenopausal women [17]. However, further studies with a larger and more inclusive population should be carried out to investigate this linkage in women before menopause. Certain enteric bacteria, whose genome is called the estrobolome, metabolize estrogens [17,19]. To be excreted, estrogens are hepatically conjugated through glucuronidation or sulfonation [19]. These estrobolome bacterial species with their ß-glucuronidase activity can deconjugate excreted estrogens in the bile and prevent their excretion [17,19]. This may explain why fecal glucuronidase levels were reported to be inversely associated with gut estrogen levels [18]. The human gut also carries out various local and distant functions through hormonal metabolites and intermediates [19]. Remarkably, gut microbes also carry out the synthesis of estrogen-like compounds from nutrition [17]. In healthy females, probiotic administration has shown promise. While probiotic administration fails to engender persistent gut microbiota changes, it has been reported to improve vaginal lactobacilli concentration, female health system bowel movement, and immune system responses in healthy adults [20]. Moreover, probiotics help enhance local vaginal immunity and maintain female reproductive tract health [21]. Notably, certain bacterial strains, namely, *Lactobacillus* strains have been shown to prevent the recurrence of urinary tract infections and bacterial vaginosis [22,23].

## 3. Gut Microbiome and PCOS

PCOS affects 8–13% of women worldwide in their reproductive age [24]. While the clinical phenotype may vary, insulin resistance and hyperandrogenism are the hallmarks of PCOS [25]. The gut microbiome of PCOS sufferers differs from that of controls [26]. As can be seen in Table 1 and Figure 1, PCOS patients have a less diverse gut microbiome, which is correlated with hyperandrogenism [25,27,28]. In a clinical trial, 43 healthy females and 50 PCOS patients were employed, taking into consideration the influence of body weight [26]. While Alpha (α) diversity was comparable in both groups, Beta (β) diversity, a measure of diversity among different samples, was markedly decreased in the gut microbiota of PCOS patients [15,26]. Conversely, a study reports that α diversity is altered in PCOS patients’ guts [29]. Another study confirms the alteration by reporting a decrease in α diversity in the guts of women with PCOS [30]. Notably, the abundance of a Bacteroides species responsible for the deconjugation of conjugated bile acids synthesized in the liver, *Bacteroides vulgatus* (*B. vulgatus*), was significantly higher in women with PCOS than in the controls [26]. Firmicutes, a phylum correlated with obesity, is also more abundant in PCOS patients while *Tenericutes ML615J-28*, *Tenericutes 124-7*, and *Bacteroidetes S24-7* levels are reduced [25,27,28]. An increase in *streptococcus* and the ratio of *Escherichia/Shigella* was also reported in the gut of women with PCOS [25]. Opposingly, it was found that *Akkermansia,* a species reported to modulate energy metabolism and glucose tolerance in humans, and *Ruminococcaceae,* were less abundant in PCOS patients [25,31].

Some clinical trials imply a lack of association between the disruption of the estrous cycle and microbiome in PCOS [48]. However, studies have reported that the gut microbiome is involved in the clinical manifestation of PCOS [28,49,50]. Murine studies report that gut microbiome indeed alters female physiology. After oral–fecal transplantation from the gut microbiota of PCOS individuals, mice exhibited insulin resistance, disrupted estrous cycle, higher number of cyst-like follicles, fewer corpora lutea, and elevated testosterone and luteinizing hormone than the controls [26]. Remarkably, these mice produced fewer pups after the oral lavage [26]. In humans, it was reported that the serum insulin level in PCOS patients was markedly correlated with various intestinal bacteria including Collinsella [25]. A disordered gut microbiome is correlated with obesity and more than half of PCOS patients are obese [28]. Consequently, correlations between obesity associated with PCOS and the gut microbiome were described. In a recent clinical trial, [25] it was reported that the abundance of *Tenericutes* in obese PCOS patients and control obese individuals was comparable. However, β diversity differences have been reported in non-obese and obese PCOS patients, which were not observed between obese and non-obese control individuals [28]. Thus, gut microbiome disturbances may be a factor in obesity specifically associated with PCOS.

There are various mechanisms behind the gut microbes’ participation in the clinical course of PCOS. Bile acids endorse the digestion and absorption of fat-soluble substances [28]. However, bacteria are crucial to bile acid transformation [28]. The gut microbiome influences host metabolism through interactions with host signaling pathways [26,29]. For instance, changes in the microbiome of PCOS patients affect bile acid metabolism [26,29]. Specifically, levels of the bile acids: glycodeoxycholic acid (GDCA) and tauroursodeoxycholic acid (TUDCA) were decreased in the stool and serum of PCOS patients [26]. Remarkably, the abundance of *B. vulgatus* was negatively correlated with that of GDCA and TUDCA [26]. Furthermore, *B. vulgatus* species in PCOS patients exhibited a higher abundance of bile salt hydrolase genes than controls [26]. Hence, the gut microbiome influences its host metabolism.

Through the decomposition of organic material, gut microbes produce short-chain fatty acids (SCFA) and other metabolites to supply the host with energy [28]. SCFAs regulate glucose uptake and fatty acid oxidation through activating peroxisome proliferator-activated receptor gamma (PPAR-γ) in the liver and muscles [28]. Notably, SCFAs bind to free fatty acid receptors FFAR-2 and FFAR-3 leading to the inhibition of appetite stimulating hormone [28]. This inhibition hinders the secretion and transformation of sex hormones, specifically the transformation of androgen to estrogen [28]. Secondly, gut microbes can affect insulin sensitivity through branched short amino acids (BCAA). An increase in BCAAs has been associated with the development of diabetes type 2 [24,28]. Gut bacteria from the genus *provotella* are BCAAs synthesizers [28]. Thus, an increase in these bacteria may lead to insulin resistance. In [25] it is reported that the gut microbiome of obese PCOS patients employed in their clinical trial harbored *Paraprevotella* and *Alloprevotella*.

Lipopolysaccharides (LPS) are a cell wall constituent in Gram-negative bacteria such as Bacteroides [28]. After their absorbance to the blood, LPS can bind to Toll-like receptor 4 and activate signaling pathways that affect insulin sensitivity and, ultimately, lead to insulin resistance [28]. After being fermented by the gut microbiota to trimethylamine, the nutrient choline is hepatically metabolized to trimethylamine oxide (TMAO), an osmotic substance involved in regulating insulin resistance [28]. Thus, an increase in trimethylamine-producing bacteria leads to higher TMAO levels, which may aggravate PCOS [28]. Further work is warranted to elucidate the precise role of the gut microbiome in PCOS. Future studies should be carried out to develop therapies that modulate the gut microbiome in order to alleviate or prevent PCOS.

## 4. Gut Microbiome and Cancer

Disturbances in the gut microbiome have been associated with and observed in various cancers including gastric, colorectal, hepatic, pancreatic, and prostate cancer [51,52,53]. Interestingly, in pancreatic cancer and melanoma murine models, a significant decrease in subcutaneous tumor burden was observed after depleting the gut microbiome through antibiotic administration [51]. Dysbiosis of the gut microbiota is also observed in various cancers affecting the female population including breast, cervical, and ovarian cancer as depicted in Table 1 and Figure 1 [45,46,47]. Furthermore, some studies imply a decrease in the diversity of the gut microbiota and an increase in the abundance of *Clostridiales* in breast cancer patients [45]. An analysis of the gut microbiota of postmenopausal women with breast cancer reveals that while the differences in relative species abundance in gut microbiota between premenopausal breast cancer patients and premenopausal controls was negligible, 45 species differed significantly in their relative abundance between postmenopausal patients and postmenopausal controls [54]. Moreover, in postmenopausal cancer patients, 38 species were overrepresented such as *Escherichia coli, Actinomyces* sp*. HPA0247, Klebsiella* sp*_1_1_55, Prevotella amnii,* and *Shewanella.* A study revealed that α and β diversity differed significantly between employed cervical cancer patients and cancer-free women [47]. Additionally, the study disclosed that cervical cancer patients had higher levels of *Prevotella, Porphyromonas,* and *Dialister* while cancer-free individuals had greater levels of *Bacteroides, Alistipes,* and members of the *Lachnospiracea* than controls [47]. In ovarian cancer patients, a shared increase in *Prevotella* regardless of platinum sensitivity was observed, but the study was unable to count for the potential effects of chemotherapy [46].

Aside from regulating the host’s immune system, the gut microbiome is involved in both oncogenesis and the suppression of malignant transformation [51,55]. Furthermore, bacterial metabolites produced by the gut microbiome also regulate cancer cell metabolism [45,56]. These secreted bacterial metabolites act like hormones since they can enter the circulation, reach far targets, and carry out important functions such as impacting mitochondrial metabolism and modulating the behavior of breast cancer cells, lithocholic acid (LCA), SCFAs, cadaverine, and deconjugated estrogens [45,56]. As mentioned in the previous section, the gut microbiome is an important player in estrogen metabolism. Since 80% of breast cancer cases are estrogen receptor-positive, the deconjugation of estrogens by the gut microbiome is of relevance [19,57,58,59,60,61]. Aside from being more abundant in the gut of breast cancer patients, *Clostridiales* reactivates estrogens and increases their serum levels [45]. Estrogen receptors play a direct role in the expression of nuclear-coded mitochondrial proteins [45]. An increase in oxidative phosphorylation promotes metastasis [45]. The gut microbiome also synthesizes estrogen-like compounds from dietary sources [17].

The gut microbiome generates the SCFAs: formate, acetate, propionate, butyrate, and lactate, through the fermentation of non-digestible carbohydrates and in minute amounts through amino acid degradation [45]. With receptors on cancer and stromal cells, SCFAs regulate various cancer hallmarks such as cell proliferation, gene expression, cell invasion, apoptosis, and metabolism in breast cancer [45]. SCFAs in general can serve as energy substrates for cancer cells [45]. Interestingly, sodium butyrate drives oxygen consumption in breast cancer cell lines and inhibits lactate metabolism, markedly decreasing breast cancer cell viability [45]. The anaerobic bacteria *Clostridiales* are mainly responsible for bile acid transformation [45]. At the serum level, the secondary bile acid LCA exhibits an antineoplastic effect on breast cancer cells, regulates oxidative phosphorylation, and inhibits proliferation [45]. Remarkably, the gut microbiota of breast cancer patients displayed a reduced ability to synthesize LCA [45]. Gut bacteria including Shigella flexneri, *Shigella sonnei*, *Escherichia coli*, and *Streptococci* carry out the biosynthesis of cadaverine from lysine [45]. Cadaverine is important because it hinders cell proliferation and tumor infiltration [45]. Interestingly, cadaverine was ineffective on primary untransformed cells and the microbiome’s capacity to synthesize cadaverine is decreased in breast cancer patients [45]. The link between the gut microbiota and gynecological cancers requires further study [62] However, just like breast cancer, ovarian cancer is correlated with estrogen abnormalities [62]. In cervical cancer patients, gut microbiota profiles and β diversity differed markedly from cancer-free women [63]. Namely, the *Proteobacteria* phylum was significantly more abundant in cervical cancer patients [63]. Thus, gut microbial modulation should be considered as a therapeutic option or at the very least an additional therapeutic strategy in tandem with traditional cancer therapies.

## 5. Gut Microbiome and Pregnancy

Aside from infant health, evidence indicates that maternal microbiome niches influence maternal well-being and post-partum recovery [64]. Furthermore, gut microbiome disturbances have been linked with the clinical characteristics of preeclampsia, a pregnancy complication characterized by high blood pressure, and some even hypothesize that there is a link between the maternal gut microbiome and postpartum depression [64,65]. In fact, a Chinese herbal medicine has been shown to ameliorate postpartum depression through modulating the gut microbiota [66]. A study also indicates that the maternal gut microbiome may play a part in the immunological adaptations accompanying pregnancy, as shown in Table 1 and Figure 1 [67]. Interestingly, an investigation of gut microbiota changes in patients with positive immune antibody-associated recurrent miscarriage reveals that some highly abundant genera, such as *Blautia* and *Bacteroides*, may be incriminated in recurrent miscarriage [37]. Bidirectional interactions between the gut microbiome and pregnancy have been reported. For instance, bacterial growth can be influenced by hormonal changes [68,69]. Moreover, gut microbial changes during pregnancy are mediated by hormonal changes accompanying gestation [33,34]. Namely, it has been reported that fecal progesterone levels were negatively correlated with diversity during pregnancy [34]. Notably, profound alterations in the microbial profile of the gut microbiome have been observed during the progression of pregnancy such as an increase in *Actinobacteria, Proteobacteria,* and opportunistic pathogens, and a decrease in SCFA producers and in overall species richness [35,36].

In humans, an analysis of the gut microbiome of thirty-five women in their first and third trimesters of pregnancy reveals that *Bifidobacterium, Blautia*, unclassified *Ruminococcaceae*, *Bacteroides*, unclassified *Lachnospiraceae*, unclassified *Clostridiales, Akkermansia, Faecalibacterium, Ruminococcus,* and *Prevotella* were the generally dominant bacterial species. Interestingly, *Bifidobacterium* is crucial for human milk oligosaccharide degradation and *Prevotella* metabolizes estradiol and progesterone [36]. Differences were also observed between the two semesters. Furthermore, *Bifidobacterium, Neisseria, Blautia,* and *Collinsella* increased most significantly in the third semester while *Dehalobacterium, Clostridium,* and *Bacteroidales* were markedly higher in the first [36]. Another report disclosed that maternal microbiome biodiversity changes with the progression of pregnancy and is associated with gestational weight gain [70]. While studies imply that gut microbiota changes dramatically such as an increase in lactic acid-producing bacteria coupled with a decrease in butyrate-producing bacteria, a recent analysis conducted on Japanese women during early and late pregnancy negates differences between late and early pregnancy microbial composition and reveals that the recruited women did not show notable differences in gut microbiota related to pregnancy, except for the phylum TM7, which decreased in late pregnancy [71,72]. Similarly, another study confirms a lack of difference and mentions that the study carried out by [35], which reported significant changes associated with pregnancy, recruited women who were consuming probiotic supplementation [73]. Interestingly, studies imply that pregnancy-induced changes in the female gut microbiome occurring at the onset of pregnancy may be vulnerable to modulation by diet while being independent of maternal weight gain and even the number of successive pregnancies [74,75]. Thus, in late pregnancy, the microbiota readjusts carbohydrate-related functions expression in consistency with the high glucose availability [76]. Notably, the microbiome of pregnant women can also bring about metabolic alterations in germ-free hosts. Furthermore, a study disclosed that fecal transplantation from pregnant women to germ-free mice induced greater adiposity and insulin insensitivity [35]. Given the plethora of studies indicative of the effect of the gut microbiome in pregnancy, further work is warranted to comprehend a more detailed mechanistic understanding as well as work to develop pre/pro/postbiotics for pregnant women.

Similarly, animal studies reported changes in gut microbiota accompanying pregnancy. In pigs, different stages of pregnancy brought about distinct changes in the abundance of *Tenericutes, Fibrobacteres,* and *Cyanobacteria* [33]. Overall, the α diversity values of the gut microbiota and the abundance of *Clostridiales, Desulfovibrio,* Mogibacteriaceae, and *Prevotella* increased over the course of pregnancy only to decrease at weaning [33]. The progression of pregnancy markedly affected the beta diversity of the gut microbiota and modified the abundance of multiple carbohydrate-degradation bacteria: *Bacteroides, Prevotella, Parabacteroides,* and *Succinivibrio* [33]. Another study conducted on sows reveals changes in the gut microbiota across the perinatal period with variance in microbial function and abundance between the prenatal and postnatal periods where the alpha diversity was higher in the latter [77]. Furthermore, *Akkermansia*, *Desulfovibrio*, *Methanobrevibacter,* and *Turicibacte*r were enriched in the prenatal period while *Actinobacillus*, *Acidaminococcus*, *Megasphaera*, *Eubacterium*, *Butyricimonas*, *Paludibacter*, *Rummeliibacillus*, and *Succiniclasticum* were enriched in the postnatal period. The changes in gut microbiota accompanying cow parturition were investigated through 16S rRNA and metagenomic sequencing, revealing notable changes in the gut microbiome throughout the late pregnancy to the postpartum stage [78]. During late pregnancy, *Lactobacillus*, *Streptococcus*, and *Clostridium* were enriched while *Bacteroides, Escherichia*, and *Campylobacter* were more abundant at postpartum [78]. Similarly, a study reports substantial remodeling of sow gut microbiota during the late stages of pregnancy to the postpartum stage [69]. Furthermore, the gut bacterial richness of both pregnant and delivery sows decreased markedly while the β-diversity notably expanded [69]. The relative abundance of *Lactobacillus* notably increased from the late pregnancy to the postpartum stage while the *Bacteroidetes* to *Firmicutes* ratio and the relative *Prevotella* abundance decreased [69]. An investigation of gut microbiota composition alterations across different reproductive periods of Tibetan macaques wild females revealed nonnegligible deviations in taxonomic structure, composition, and potential functions of gut microbes [79]. The study revealed an increase in the relative abundance of *Proteobacteria* during pregnancy and lactation, and an overrepresentation of the relative abundance of Succinivibrionaceae and Bifidobacteriaceae in pregnant and lactating females, respectively [79].

## 6. Changes in Gut Microbiome during the Menstrual Cycle

The menstrual cycle extends 28 ±  4 days and is comprised of the follicular, luteal, and menstrual phases [80,81]. The menstrual cycle is accompanied by significant hormonal fluctuations. Moreover, the level of the steroid hormone estrogen soars in the middle of the follicular phase, drops after ovulation, and rises back again in the early luteal phase [80]. The early luteal phase is also characterized by an increase in the progesterone level [81]. The sudden drop in these two hormones in the late luteal phase brings about menses [81]. Many healthy females report variations in gastrointestinal (GI) symptoms during their menstrual cycle, which may be deduced to the presence of sex hormone receptors along the GI tract [81]. For instance, a study investigating the relationship between menstrual cycle phase, daily stool number, and consistency reveals looser stool consistency in the early menstrual period in comparison to midcycle in six out of the seven employed participants [82]. However, studies investigating GI transit throughout the menstrual cycle produce contradicting results. Furthermore, some disclose an increase in transit time during the luteal phase, accompanying the increased progesterone levels while others negate any change [13,83,84].

Hormonal fluctuations may impose pressures on the function and composition of the human microbiome [85]. Namely, the gut microbiome is reported to be influenced by estrogen [86]. Furthermore, estrogen levels are associated with gut microbiome alpha diversity and fecal *Clostrdia* taxa [13]. Given the influence estrogen has on the gut microbiome, the potential link between the GI disturbances and menstruation may potentially be mediated by the gut microbiome. The gut microbiota is also reported to be affected by the steroid hormone progesterone. A study reports the amelioration of depression and anxiety-like behaviors accompanying the premenstrual, post-partum, and premenopausal periods by progesterone in mice [84]. However, that effect was undermined by antibiotic treatment, indicating that this improvement is mediated through progesterone’s enhancing effect on *Lactobacillus reuteri* growth [84].

The influence between sex hormones and the gut microbiome is bidirectional. Moreover, bacteria can metabolize sex hormones through various enzymes such as hydroxysteroid dehydrogenase, regulating the balance between active and inactive steroids [80]. Namely, fecal bacteria carry out hydrolytic reductive and oxidative reactions of androgens and estrogen [80]. Furthermore, the gut microbiome markedly influences estrogen levels [86]. This is through the gut microbiome’s secretion of β-glucuronidase, which is the enzyme responsible for estrogen deconjugation [86]. A decrease in the gut microbiome diversity affects β-glucuronidase activity adversely, lowering estrogen levels [13]. Since estrogen is only biologically active if deconjugated, this deconjugation enables estrogen to bind to its receptors: estrogen receptor alpha (ERα) and estrogen receptor beta (ERβ) [86,87]. Estrogen is crucial for homeostasis in healthy premenopausal women and its decrease accompanying menopause drives metabolic rate reduction and weight gain, yet it also stimulates epithelial proliferation within the female reproductive tract, driving various proliferative diseases such as uterine fibroids and endometriosis [86,87]. This engendered the hypothesis that the gut microbiome of endometriosis patients may have higher densities of β-glucuronidase producing bacteria than the controls [86]. In fact, a study reported that gut microbiota alterations were observed in the rhesus monkey model of endometriosis, namely, fewer *Lactobacilli* shedding in feces [88]. Interestingly, cyclic alterations in the gut microbiome may be linked with premenstrual syndrome, but this hypothesis is still under study and future work needs to be accomplished [89]. Thus, while research is required to confirm this speculation, we hypothesize that alterations in the gut microbiome may potentially bring about menstruation-related diseases.

There is a scarcity of studies investigating the relationship between the hormonal fluctuations accompanying the menstrual cycle and the gut microbiota [32]. However, studies investigating the relationship between menstruation and other human microbiomes have been performed. A study reports a lack of significant menstruation-driven changes in the saliva and fecal microbiomes, yet discloses an increased diversity in the vaginal microbiome during menses, which is ensured by an expansion of *Lactobacillus* during the follicular and luteal phases [90]. Similarly, another recent study reports increased vaginal microbial diversity and a correlation between *Lactobacillus* abundances and predicted estradiol levels across the menstrual cycle [91]. Analyses of the oral microbiome were also carried out. Interestingly, anaerobic bacterial counts in saliva are reported to increase during ovulation [85]. An analysis of the salivary profile of 309 women in their reproductive age during the menstrual, follicular, and luteal phases of the cycle reports a lack of significant differences in α-diversity or phase-specific clustering of the overall microbiome but discloses variance in the abundances of *Prevotella*, *Campylobacter, Haemophilus,* and *Oribacterium* throughout the cycle, where a higher species-richness was noted in the luteal phase [85].

## 7. Gut Microbiota Composition Alterations Accompanying Menopause

Female sex hormones such as estrogen impact microbiota in various body sites, especially the gut [16]. When women possess sufficient estrogen, their gut microbiota displays species diversity where beneficial bacteria are dominant and harmful bacteria growth is inhibited [38]. The gut microbiome has been correlated with menopause: the cessation of menstruation accompanied by estrogen down-regulation, ovary function loss, and hormone receptors dysfunction [16,38]. Menopause is associated with a lower gut microbial species diversity [92]. Thus, there are marked differences in the gut microbiomes and their metabolites in premenopausal and postmenopausal women, as shown in Table 1 and Figure 1 [93]. Changes in the gut microbiome have been reported in the perimenopausal period, the period before menopause occurs. Namely, during the perimenopausal period, the relative abundance of beneficial bacteria such as *Lactobacillus* and *Bifidobacteria* is markedly reduced while that of harmful bacteria such as *Enterobacter* is increased in women [38]. In a study, bilateral ovariectomizing was employed to investigate gut microbiota changes accompanying perimenopause and it revealed that ovariectomized mice displayed the lowest abundances, which was regulated by estrogen supplementation, implying a bidirectional relationship between the microbiota and estrogen [38]. Moreover, the study discloses that obesity in peri- and post-menopausal women is associated with possessing a gut microbiota unable to metabolize the soy isoflavone daidzein to *O-*desmethylangolensin [94].

Of note, the gut microbiota of post-menopausal women was observed to be closer in resemblance to men than that of pre-menopausal women [39]. Namely, postmenopausal women, similar to age-matched men, have a lower abundance of SCFA-producing bacteria [39]. Furthermore, the number of species from genera that differentiate men from women decreased after menopause implying a masculinization of the gut microbiota composition postmenopause [39]. Studies report that premenopausal women have higher abundances of several *Alistipes*, *Bifidobacterium*, and *Ruminococcus* species and lower abundances of *Bacteroides*, *Prevotella*, and *Haemophilus* species, while postmenopausal women have fewer *Firmicutes* and *Roseburia* spp., and more *Bacteroidetes* and *Tolumonasare* in their fecal samples [39,93]. Furthermore, the ratio of *Firmzicute*s/*Bacteroides*, and the relative abundances of *Lachnospira* and *Roseburia*, are elevated in the gut microbiota of postmenopausal women, while the relative abundances of *Prevotella, Parabacteroides,* and *Bilophila* are reduced [38]. Another study reported a higher *Firmicutes/Bacteroidetes* ratio and relative abundance of *Lachnospira* and *Roseburia*, but a lower relative abundance of the *Prevotella*, *Parabacteroides,* and *Bilophila* genera in pre-menopausal women than in post-menopausal women [40]. However, aside from β diversity, no differences of significance were reported in alpha diversity indices among pre- and post-menopausal women [39]. With its secretion of β-glucuronidase, the enzyme responsible for estrogen’s deconjugation into its active forms, the gut microbiome can affect the circulating levels of estrogens [86]. Moreover, dysbiosis of the gut microbiome, leading to lower microbial diversity, may decrease circulating estrogen as its deconjugation would be reduced [86]. Interestingly, the administration of a novel strain (YT2) of *Lactobacillus intestinalis* was found to be significantly reduced in ovariectomized rats, which led to a marked amelioration of menopausal symptoms such as increased fat mass, decreased bone mineral density, and remarkably, it also restored the intestinal microbial composition and increased *Firmicutes*/*Bacteroides* ratio [95]. Nonetheless, studies to determine the efficacy of these therapies in clinical trials are needed.

## 8. The Role of the Gut Microbiome in Postmenopausal Female Health

The gut microbiome has been correlated with various diseases accompanying menopause. Obesity affects 65% of postmenopausal women and interestingly, the relationship between the gut microbiota and estrogen is speculated to mediate this weight gain [16]. Moreover, the gut microbiome has been related to obesity, and menopause is associated with a heightened risk of obesity [92,93]. Notably, other than the differences in *Akkermansia muciniphila*, *Bifidobacterium animalis, Dorea,* and *Desulfovibrio,* the gut microbial characteristics of diet-induced and bilaterally ovariectomized obese mice are reportedly similar [38]. However, a study reports that while menopausal obesity and dietary obesity led to similar gut microbiome structures, menopausal obesity engenders a different intestinal microbiota, namely, *Bifidobacterium animalis,* which was solely observed in the ovariectomized mice [96].

Notably, the gut microbiome was reported to impact skeletal muscle mass through its synthesis of SCFA butyrate in healthy menopausal women [97]. Moreover, increased capacity for gut microbial synthesis was markedly associated with serum butyrate levels and skeletal muscle index, and two main butyrate-producing bacterial species, *Faecalibacterium prausnitzii,* and *Butyricimonas virosa,* were positively associated with this increased capacity for gut microbial synthesis of butyrate and the skeletal muscle index [97]. Gut ecology was also reported to contribute to the mediation of the protective effects increased aerobic capacity may have against menopause-associated cardiometabolic risk, especially the production of signaling molecules such as short-chain fatty acids produced by the gut [98]. Moreover, another study reports that the response to physical exercise, which is reported to modify the intestinal microbiota composition, is actually contingent upon the initial microbiota profile [92].

Postmenopausal women with breast cancer have been reported to possess an altered composition and estrogen-independent low diversity of their microbiota [16,54]. An analysis reports that postmenopausal women recently diagnosed with breast cancer had a less diverse fecal microbiota with a composition that differs from that of postmenopausal women without breast cancer and higher urinary estrogens [99]. This was confirmed by another study reporting the potentially decreased postmenopausal breast cancer risk for women who possess high intestinal microbial diversity [100]. Additionally, the abundance of SCFA-producing bacteria was reduced in healthy premenopausal women while *Pediococcus* and *Desulfovibrio* were relatively characteristic of premenopausal breast cancer patients [101]. Thirty-eight species were increased in postmenopausal breast cancer patients, namely, *Shewanella putrefaciens, Enterococcus gallinarum, Escherichia coli, Klebsiella *sp_1_1_55*, Prevotella amnii, Actinomyces* sp. HPA0247*,* and *Erwinia amylovora,* while seven species were underrepresented such as *Eubacterium eligens* and *Lactobacillus vaginalis* [54].

The risk of Alzheimer’s disease is also reported to increase during the menopausal transition [96]. Interestingly, postmenopausal women make up over 60% of all Alzheimer’s patients [102]. The pathophysiology of AD commences 10–20 years before symptoms can be detected clinically, corresponding with the hormonal transitions accompanying the menopausal transition, in which many of the symptoms are risk factors themselves for AD [102]. In fact, a nonnegligible body of data reported the neuroprotective effects of estrogen and its modulatory role in female cognitive aging [102]. Thus, the prevalent complaints of cognitive decline by menopausal women are plausible given that many regions vulnerable to AD display significant overlap with the brain estrogen network [102]. As explained previously, the gut microbiota, through its deconjugation of estrogens in the bile, plays a role in determining systemic estrogen levels [17,19]. Hence, since the cognitive decline accompanying AD is largely correlated with that of estrogen, we can speculate that the gut microbiome may mediate or contribute to the deflation in estrogen systemic levels in menopausal women. Notably, studies utilizing a murine model disclose that gut dysbiosis may be a risk factor for AD [103,104]. Moreover, using an AD-like pathology with amyloid and neurofibrillary tangles (ADLP^APT^) transgenic mouse model of AD, a study reports differences in the gut microbiota composition of healthy wild-type mice and that of ADLP^APT^ [103]. Additionally, ADLP^APT^ mice displayed a loss of epithelial barrier integrity and chronic intestinal and systemic inflammation [103]. Notably, transplantation of the fecal microbiota from wild-type mice into ADLP^APT^ mice ameliorated the formation of neurofibrillary tangles, amyloid β plaques, glial reactivity, and cognitive impairment [103].

## 9. The Role of the Gut Microbiome and Postmenopausal Bone Health

Among postmenopausal women, osteoporosis and its precursor osteopenia are prevalent metabolic bone diseases [41]. The gut microbiome has also been implicated in bone-related diseases among postmenopausal women and their manifestations, as shown in Table 1 and Figure 1. Moreover, an increase in gut permeability, which is associated with lower bone mineral density, has been reported during perimenopause [105]. A study reports decreased bacterial richness and diversity, and significant differences in abundance levels among phyla and genera in the gut microbial community in postmenopausal osteoporosis [42]. However, the study negates any significant correlation between bacterial diversity and estrogen [42]. An analysis of fecal samples from postmenopausal women with osteoporosis and with normal bone mass reveals a marked discrepancy between the gut microbiota of both groups [43]. Namely, the proportion of the genus *Prevotella* was notably higher in postmenopausal women with normal bone mass, implying a potential bone-protective effect of *Prevotella* [43]. Moreover, fracture incidence was markedly higher in postmenopausal women with low *Bacteroides* abundance than in controls [44]. Conversely, a recent study reports that *Bacteroides* were more prevalent in osteoporosis and osteopenia groups [41]. That study also reveals significant taxonomic compositional differences in osteoporotic and osteopenic, and healthy postmenopausal women, such as a higher abundance of unclassified *Clostridia* and methanogenic archaea, than in healthy postmenopausal women [41]. Another study recognized taxa-specific variations in the intestinal microbiota associated with bone turnover markers, especially C-terminal cross-linking telopeptide of type I collagen (CTX) [106]. This could be elucidated by the hormonal changes characterizing menopause. Furthermore, the lack of female hormones brings about bone loss and osteoporosis [96]. Interestingly, probiotic administration was reported to ameliorate osteopenia in postmenopausal women. Moreover, the administration of probiotic treatment and bioavailable isoflavone attenuated bone mineral density loss brought about by estrogen deficiency, promoted a favorable estrogen metabolite profile, and improved bone turnover [107].

## 10. Therapeutic Strategies to Modulate the Gut Microbiome in Females

In light of reported gut microbial compositional changes accompanying various female diseases, investigating the therapeutic potential of probiotic administration or fecal transplant is suggested. Moreover, the female diseases we discussed were reported to be accompanied by gut dysbiosis. Hence, probiotic administration of certain bacterial species to correct a deficiency or an overgrowth may be of therapeutic value. Firstly, PCOS patients are reported to have a less diverse gut microbiome, specifically lower β diversity than controls [25,26,27,28]. Notably, the abundance of certain bacterial species was shown to be reduced while that of others was enhanced. Furthermore, the abundance of *Bacteroides vulgatus* (*B. vulgatus*), *streptococcus,* ratio of *Escherichia/Shigella*, and Firmicutes is higher, while that of *Tenericutes* ML615J-28, *Tenericutes* 124-7, Ruminococcaceae, Akkermansia, and *Bacteroidetes* S24-7 is lower in PCOS patients than the controls [25,27,28]. Hence, the administration of the latter species may ameliorate the condition. In fact, the administration of vitamin-D in conjugation with a probiotic supplement containing *Lactobacillus acidophilus*, *Bifidobacterium bifidum, Lactobacillus reuteri,* and *Lactobacillus fermentum* for 12 weeks was shown to ameliorate some of PCOS symptoms [108]. Similarly, the administration of selenium with a probiotic supplement consisting of *Lactobacillus acidophilus*, *Lactobacillus reuteri*, *Lactobacillus fermentum,* and *Bifidobacterium* bifidum led to the same results [109]. Secondly, various cancers affecting the female population, such as breast, cervical, and ovarian cancer, were associated with gut microbiota alterations [45,46,47]. Namely, breast cancer patients were reported to display a heightened abundance of *Clostridiales* in breast cancer, cervical cancer patients had higher levels of *Prevotella, Porphyromonas,* and *Dialister* and lower levels of *Bacteroides, Alistipes,* and members of *Lachnospiracea,* and ovarian cancer patients, displayed an increase in *Prevotella* [45,46,47]. Thus, a correction of these compositional deviations could be of relevance. For instance, several studies display promising evidence that diet, probiotics, and prebiotics could have an important therapeutic effect on breast cancer [110]. Thirdly, various diseases arising during the period and the transition to menopause are associated with gut alterations. Interestingly, *Bifidobacterium animalis* was observed in a murine model of menopausal obesity [96]. Hence, an investigation of the role of this bacterial species could be carried out and if found contributing to obesity, the introduction of a competing species could be a possibility. Markedly, an administration of a probiotic mix consisting of *Bifidobacterium lactis* W51, *Bifidobacterium bifidum* W23, *Lactobacillus brevis* W63, *Bifidobacterium lactis* W52, *Lactobacillus salivarius* W24, *Lactobacillus acidophilus* W37, *Lactococcus lactis* W19, *Lactobacillus casei* W56, and *Lactococcus lactis* W58 was shown to positively affect vascular function and reduce arterial stiffness in obese postmenopausal Women [111]. Postmenopausal normal bone mass was associated with higher *Prevotella* abundance [43]. As mentioned previously, probiotic administration treatment and bioavailable isoflavone-attenuated bone mineral density loss are brought about by a deficiency in estrogen and improved bone turnover [107]. However, the administration of *Prevotella* may specifically prove protective against postmenopausal bone mass loss. Finally, studies utilizing murine models reveal that gut dysbiosis may be a risk factor for AD [103,104]. Interestingly, probiotic supplementation improved cognitive function and mood in adults above 65 years and had favorable outcomes on AD specifically [112,113].

## 11. Conclusions

Trillions of microorganisms populate the human GI tract and are known to protect the host from adversities. The gut microbiome has a profound role in the elimination of pathogens and may contribute to many diseases affecting females such as breast, cervical, and ovarian cancer, polycystic ovarian syndrome, and postmenopausal period illnesses such as menopausal obesity, Alzheimer’s disease, and bone diseases (Table 1 and Figure 1). Traditionally most research has focused on male subjects and there is a need to undertake more research on female health. A recent study, the first of its kind examined the impact of menopause on women’s metabolism, as well as diet and how this related to their overall health [114]. The study indicated that diet and gut microbial species may have been responsible for changes observed after menopause such as higher blood pressure and a greater risk of developing cardiovascular diseases. The study indicated the important contribution of the gut microbiome and diet to female health. Future work should focus on developing therapy based on an individual’s metabolic and hormonal status as well as exploring the critical role of the microbiome. The modulation of the gut microbiome via diet and through supplementation with pre/pro/postbiotics in various female health issues should be undertaken. The precise optimal composition of microbial species is not known. Furthermore, modulation of the gut microbiome as a therapeutic/preventative strategy needs to be accomplished, with a focus on female health. Further studies are essential to ascertain the metabolites/molecules produced by the gut microbiota and their effects on female-related health issues are warranted. 

## Figures and Tables

**Table 1:** The impact of gut microbiota on female health.

Condition	Dysbiosis	Strains Increased	Strains Decreased	References
Menstruation	Little is known about the relationship between menstruation and the gut microbiome.			[32]
Pregnancy	Gut microbial changes mediated by gestational hormonal changes	*Actinobacteria, Proteobacteria,* and opportunistic pathogens	SCFA producers and in overall species richness	[33,34,35,36]
positive immune antibody-associated miscarriage	Unreported	*Blautia* and *Bacteroides*	None reported	[37]
Perimenopause	Present	*Enterobacter*	*Lactobacillus* and *Bifidobacteria*	[38]
Postmenopause	Grows in resemblance to male gut microbiome	Conflicting results	Conflicting results	[38,39,40]
Postmenopause-associated bone diseases	decreased bacterial richness and diversity	unclassified *Clostridia* and methanogenic archaea	*Prevotella*Studies report conflicting results on *Bacteroides*	[41,42,43,44]
PCOS	Present with decreased diversity	*Bacteroides vulgatus, Firmicutes, Streptococcus,* and the ratio of *Escherichia/Shigella*	*Tenericutes ML615J-28*, *Tenericutes 124-7*, *Akkermansia, Ruminococcaceae,* and *Bacteroidetes S24-7*	[25,26,27,28,30,31]
Breast cancer	Present with a decrease in diversity	*Clostridiales*	None reported	[45]
Ovarian cancer	Present	*Prevotella,* yet the effects of chemotherapy have not been accounted for.	None reported	[46]
Cervical cancer	Present with changes in diversity	*Proteobacteria, Prevotella, Porphyromonas,* and *Dialister*	*Bacteroides, Alistipes*, and members of the *Lachnospiracea*	[47]

## Data Availability

Not applicable.
